# An emerging new fowl adenovirus genotype

**DOI:** 10.1016/j.heliyon.2019.e01732

**Published:** 2019-05-25

**Authors:** Győző L. Kaján, Ilaria Affranio, Andrea Tóthné Bistyák, Sándor Kecskeméti, Mária Benkő

**Affiliations:** a Institute for Veterinary Medical Research, Centre for Agricultural Research, Hungarian Academy of Sciences, Hungária krt. 21, H-1143, Budapest, Hungary; b Veterinary Diagnostic Directorate, National Food Chain Safety Office, Bornemissza u. 3-7, H-4031, Debrecen, Hungary

**Keywords:** Agriculture, Virology

## Abstract

In this work, we examined the diversity of fowl adenovirus (FAdV) types occurring in Hungary. From diseased chicken flocks in Eastern Hungary, 29 FAdV strains were isolated between 2011 and 2015. We performed molecular typing of the isolates based on their partial hexon sequences. The results showed that representatives from every FAdV species from A to E are present in Hungary, but compared to the findings from our previous survey, a lower number of different FAdV types were detected. Inclusion body hepatitis was always associated with FAdV-2 or -8b, gizzard erosion was caused in almost every case by FAdV-1. Numerous strains belonging to species FAdV-B were found. The complete genome sequence of a candidate new genotype strain, showing the highest divergence from the reference FAdV-5, was determined using next generation sequencing. In order to provide results compatible with the serology-based type classification, multiple genomic regions, including the major antigenic determinants, of the new isolate (strain 40440-M/2015) were compared to their counterparts in the prototype FAdV-5 (strain 340) from species FAdV-B, at both nucleotide and amino acid sequence levels. In different comparative analyses, the two strains were always found to have larger divergence between each other than any two of the most closely related FAdV serotypes. This new emerging FAdV genotype is already present in Hungary and Austria, though its exact pathological role requires further investigations. The introduction of a novel FAdV (geno)type for the classification of these strains is further supported.

## 1 Introduction

Fowl adenoviruses (FAdVs) are responsible for a variety of clinical diseases that seem to have a globally increasing importance in the poultry industry nowadays (Schachner et al., 2017). Serologically different FAdV types are classified into five species, from *Fowl aviadenovirus A* to *E* within the genus *Aviadenovirus* (Harrach et al., 2011; Harrach and Kaján, 2011). Typical adenoviral diseases of chickens are inclusion body hepatitis (IBH), hepatitis-hydropericardium syndrome (HHS) and adenoviral gizzard erosion (GE) that are usually caused by specific virus types (Hess, 2013). From IBH cases, predominantly strains belonging to species FAdV-D and FAdV-E have been isolated in different countries (Nakamura et al., 2011; Ojkić et al., 2008; Schachner et al., 2016; Zadravec et al., 2011, 2013). Pathogenic strains of FAdV-4 (from species FAdV-C) are responsible for HHS outbreaks (Li et al., 2016; Mase et al., 2009; Mettifogo et al., 2014), whereas GE is associated with FAdV-1 (species FAdV-A) infection (Domanska-Blicharz et al., 2011; Kecskeméti et al., 2012; Lim et al., 2012; Thanasut et al., 2017).

Type identification of FAdVs has a relevant role in epizootiological studies, including disease-outbreak monitoring, and may contribute to the prevention of diseases, by the development of a proper vaccination strategy. In this work, our aim was to extend and update our knowledge concerning the epizootiological situation in Hungary. To this end, we isolated and characterized more than two dozens of FAdV strains from diseased chicken flocks in Hungary between 2011 and 2015. Additionally, an emerging new FAdV type, belonging to species FAdV-B was further analysed by complete genomic sequence and phylogeny reconstructions.

## 2 Materials and methods

### 2.1 Origin of the strains, screening PCR

Twenty-nine FAdV strains were isolated between 2011 and 2015 from Eastern Hungarian broiler and layer pullet flocks with various clinical signs. The carcasses were submitted for diagnostic investigation to the Veterinary Diagnostic Directorate of the National Food Chain Safety Office in Debrecen. The typing of the strains was conducted as described previously (Kaján et al., 2013). In brief, the isolation and propagation of the FAdV strains were done on freshly prepared primary chicken embryo liver cell culture. The nucleic acid was extracted from the samples and tested by a PCR that is capable of amplifying a variable region (the immunogenic determinant loop 1) from the gene of the hexon, the main capsid protein (Meulemans et al., 2001). The nucleotide (nt) sequence of the PCR products was determined using Sanger sequencing.

### 2.2 Phylogeny analyses

The derived amino acid (aa) sequences were aligned with the corresponding sequences from the FAdV reference strains using the Clustal W multiple alignment program (Larkin et al., 2007). The best evolutionary model for tree inference was predicted using ProtTest (Darriba et al., 2011) and was shown to be the Le-Gascuel aa replacement matrix (Le and Gascuel, 2008) with 4 categories of gamma-distributed rate heterogeneity. Moreover, the observed aa frequencies and the proportion of the invariant sites (LG + I+Γ4+F) were also taken into account. Phylogenetic analyses were conducted using PhyML 3.0 (Guindon and Gascuel, 2003). Clade support was assessed by using non-parametric bootstrapping with 1000 replicates. MEGA 7 was used for the visualization of phylogenetic trees (Kumar et al., 2016).

### 2.3 Genome sequencing of strain 40440-M/2015

One isolate, strain 40440-M/2015, shared only low-level sequence identity even with the most closely related serotype FAdV-5 (reference strain 340) belonging to species FAdV-B, therefore we decided to perform a complete genomic analysis. After large-scale propagation, the tissue culture was frozen and thawed three times. After low-speed clarification, the virions were concentrated from the supernatant by ultracentrifugation, and the viral DNA was isolated using a guanidine-hydrochloride based method (Dán et al., 2003). The extracted DNA was submitted for NGS on Illumina MiSeq by a commercial service provider.

Sequence assembly was performed using the Geneious 9.1.8 software (Kearse et al., 2012). The reads were first trimmed (error probability limit: 5%). The Geneious mapper was used to map the reads initially to the FAdV-5 reference genome sequence (strain 340: KC493646) (Marek et al., 2013), then to the first assembly's consensus sequence using the highest sensitivity and five iterations. The final consensus sequence was annotated based on the genome annotation of strain 340 using the Annotate & Predict function of Geneious. The annotation was checked and edited manually.

The complete genome sequence of strain 40440-M/2015 was aligned to that of strain 340. The sequence identity of the two strains was determined using a 10-bp-long sliding window in Geneious 9.1.8 (Kearse et al., 2012).

### 2.4 Phylogenetic analysis of strain 40440-M/2015

Traditionally, adenovirus serotypes have been demarcated on the basis of reciprocal serum-neutralization assays. To obtain comparable type determination, we used an imputed serological method: a pairwise-alignment-based sequence identity analysis. Using the MAFFT alignment algorithm (Katoh and Standley, 2013) in the Sequence Demarcation Tool (SDT) 1.2 (Muhire et al., 2014), we compared the strain 40440-M/2015 with every reference FAdV strain based on their complete genome sequences and also based on the derived amino acid sequences of the DNA polymerase, the penton base, the fiber knob and the hexon, the last one both in complete and the L1 region only. A joint analysis of all antigenic determinants (penton base, hexon loop 1, hexon loop 2 and fiber knob aa sequences) was also conducted in the form of a concatenate. Since members of species FAdV-A and -C possess and express two fiber genes (Chiocca et al., 1996; Marek et al., 2012), both fiber knob aa sequences of these species were included in the corresponding SDT analyses. In the fiber knob analysis, every separate fiber knob was compared to each other, whereas both fiber proteins were included in the concatenates for types FAdV-1, -4 and -10. Finally, as ORF19 sequences of strain 40440-M/2015 and 340 showed a high level of divergence, this open reading frame was analysed at both nt and aa levels.

Conventional, multiple-alignment-based evolutionary tree reconstructions were also performed for visualization of the phylogenetic relations. The analyses were performed with complete genomic DNA sequences as well as with the derived aa sequences of the entire hexon and the DNA polymerase. Multiple alignments were made using MAFFT (Katoh and Standley, 2013), and phylogenetic calculations were performed using RAxML 8.2.10 (Stamatakis, 2014) based on alignments edited in Gblocks 0.91b (Talavera and Castresana, 2007). Evolutionary model selection for the complete genome sequence alignment was performed using MEGA 7 (Kumar et al., 2016), and using RAxML for the protein alignments. The robustness of the trees was determined with a non-parametric bootstrap calculation using 1,000 repeats. Phylogenetic trees were visualized using MEGA 7 (Kumar et al., 2016), trees were rooted on the midpoint, and bootstrap values are given as percentages if they reached 75%.

## 3 Results

**Fig. 1:**
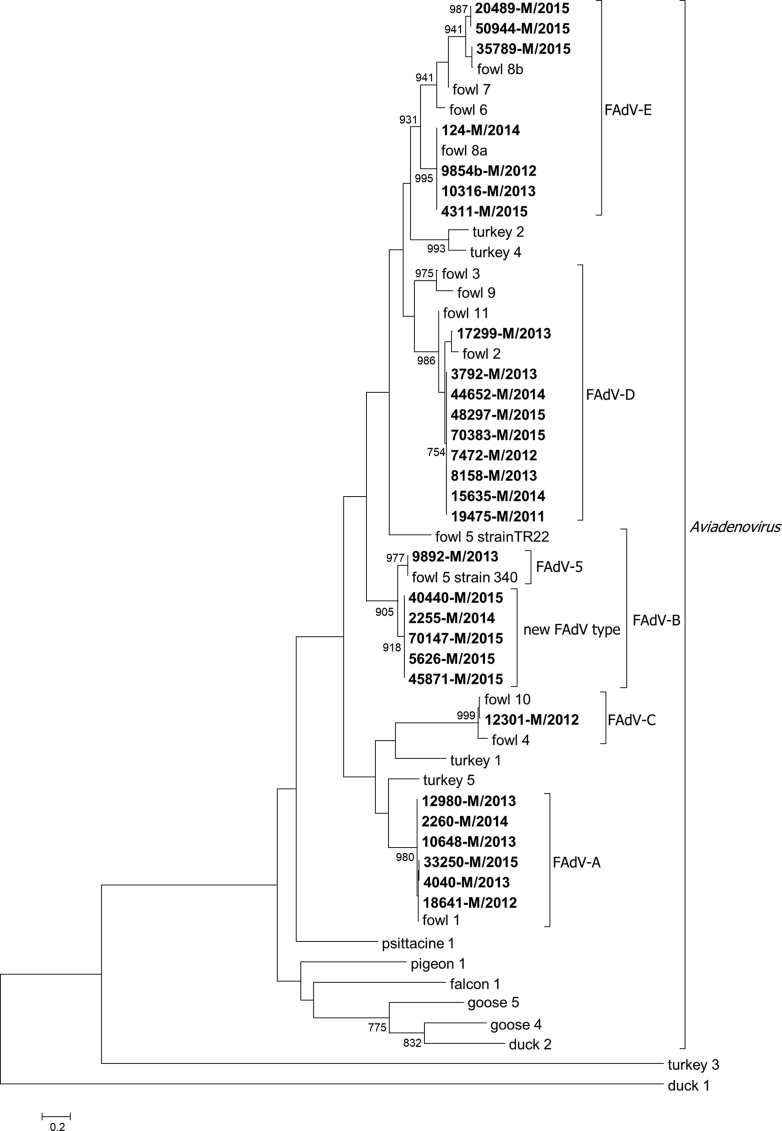
Phylogenetic analysis of the Hungarian fowl adenovirus strains based on partial derived amino acid hexon sequences. The tree was rooted on its midpoint, bootstrap values below 750 are omitted. The analysed Hungarian strains are in bold. Adenovirus types are represented by host name and type number.

The results of the molecular typing and related pathological findings are summarised in Table 1. The underlining phylogenetic tree is shown in Fig. 1. At least one isolate was obtained from all the five FAdV species. The majority of the isolates (55.2%) were classified into species FAdV-D (n = 9) and -E (n = 7). Six isolates clustered into each of species FAdV-A and FAdV-B, respectively. Only one sample (12301-M/2012) clustered into species FAdV-C. We did not observe mixed AdV infection. From the six strains that proved to fall into species FAdV-B, only one had 100% aa sequence identity with the FAdV-5 reference strain 340. The remaining five strains were identical and shared only 91.4% identity with it, based on the edited alignment of the derived aa sequences (data not shown).

**Table 1:** The classification of isolated fowl adenovirus strains grouped by the species and type of the virus.

Sp.	Type	Strain No	Pathological findings	Other positive tests
A	1	18641-M/2012	n.a.	n.a.
		4040-M/2013	anaemia, gizzard erosion, necrotising enteritis	*E. coli*
		10648-M/2013	n.a.	CAV
		12980-M/2013	fibrinous airsacculitis and pericarditis, necrotising enteritis, gut hypomotility, catarrhal enteritis, cardiac decompensation, pulmonary oedema, arthritis, acetabulum necrosis, atrophy of the gizzard and ventriculus	*E. coli*, reovirus
		2260-M/2014	cardiac decompensation, ascites, necrotising enteritis, fibrinous airsacculitis and pericarditis	*E. coli*, reovirus
		33250-M/2015	hypoxia, acute cardiac decompensation, incomplete haemostasis, pulmonary oedema, hepatic hyperaemia	*E. coli*
B	5	9892-M/2013	catarrhal enteritis, pulmonary oedema, necrotising enteritis	*E. coli*, CAV
	putative new type	2255-M/2014	cardiac decompensation, ascites, underweight, no feed uptake, exsiccosis, visceral gout	*E. coli*
		5626-M/2015	hypoxia-induced pulmonary oedema, incomplete haemostasis, catarrhal tracheitis	*E. coli*
		40440-M/2015	hypoxia, acute cardiac decompensation, incomplete haemostasis, pulmonary oedema, nephrosis	-
		45871-M/2015	fibrinous airsacculitis and pericarditis, suffocation, acute tracheitis, gut hypomotility, catarrhal enteritis, cardiac decompensation, pulmonary oedema, nephrosis	*E. coli*, IBV
		70147-M/2015	necrotising enteritis, gut hypomotility, catarrhal enteritis, cardiac decompensation, pulmonary oedema	reovirus
C	10	12301-M/2012	ascariasis, follicular rupture, peritonitis, tracheitis	-
D	2	19475-M/2011	gut hypomotility, catarrhal enteritis, cardiac decompensation, pulmonary oedema, necrotising enteritis	*E. coli*
		7472-M/2012	inclusion body hepatitis	-
		3792-M/2013	gizzard erosion, anaemia, acute cardiac decompensation, incomplete haemostasis, necrotising enteritis	*E. coli, Pseudomonas, Clostridium perfringens*
		8158-M/2013	pericardial effusion, necrotising enteritis, fibrinous airsacculitis and pericarditis, acetabulum necrosis, cardiac decompensation, suffocation, enteritis, ascites, visceral gout	*E. coli*
		17299-M/2013	necrotising enteritis, gut hypomotility, catarrhal enteritis, cardiac decompensation, pulmonary oedema, swollen bursa Fabricii, resolved infectious bursal disease	-
		15635-M/2014	no pathological findings	-
		44652-M/2014	underweight, no feed uptake, litter ingestion, cardiac decompensation, ascites, necrotising enteritis	*E. coli*, reovirus
		48297-M/2015	suffocation, acute tracheitis, coli septicaemia, gut hypomotility, catarrhal enteritis, cardiac decompensation, pulmonary oedema; no pathological findings in 4 out of 10 chickens (exterminated)	reovirus, *E. coli*
		70383-M/2015	stunted growth, dyspepsia, malabsorption, necrotising enteritis, fibrinous airsacculitis	*E. coli*
E	8a	9854-M/2012	fibrinous airsacculitis and pericarditis, necrotising enteritis, suffocation, tracheitis	*E. coli*, IBV
		10316-M/2013	n.a.	-
		124-M/2014	indigestion, litter ingestion, rachitis	reovirus and FAdV serology
		4311-M/2015	catarrhal tracheitis, hepatosis, fibrinous airsacculitis and pericarditis	*E. coli*, IBV
	8b	20489-M/2015	n.a.	reovirus
		35789-M/2015	chronic fibrinous airsacculitis and pericarditis, inclusion body hepatitis, necrotising enteritis, myocardosis	*E. coli*, *Streptococcus*
		50944-M/2015	necrotising enteritis	reovirus serology

Abbreviations: CAV: chicken anaemia virus; *E. coli*: *Escherichia coli*, FAdV: fowl adenovirus; IBV: infectious bronchitis virus; n.a.: not available; Sp.: species.

**Fig. 2:**
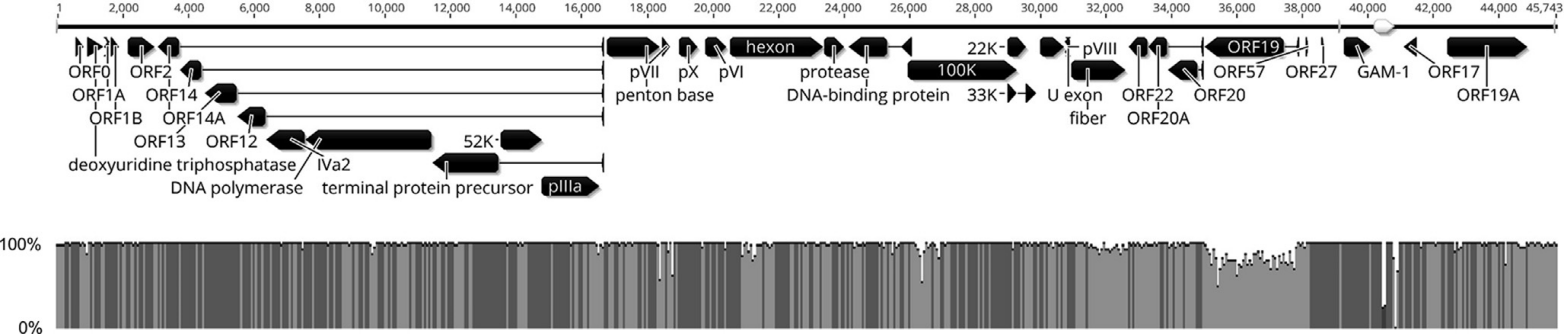
Genome layout of strain 40440-M/2015, a putative new fowl adenovirus type within species *Fowl aviadenovirus B*, and its sequence identity to strain 340 (FAdV-5, KC493646). Black arrows in the genome map represent protein coding sequences, white arrows represent repeat regions.

**Fig. 3:**
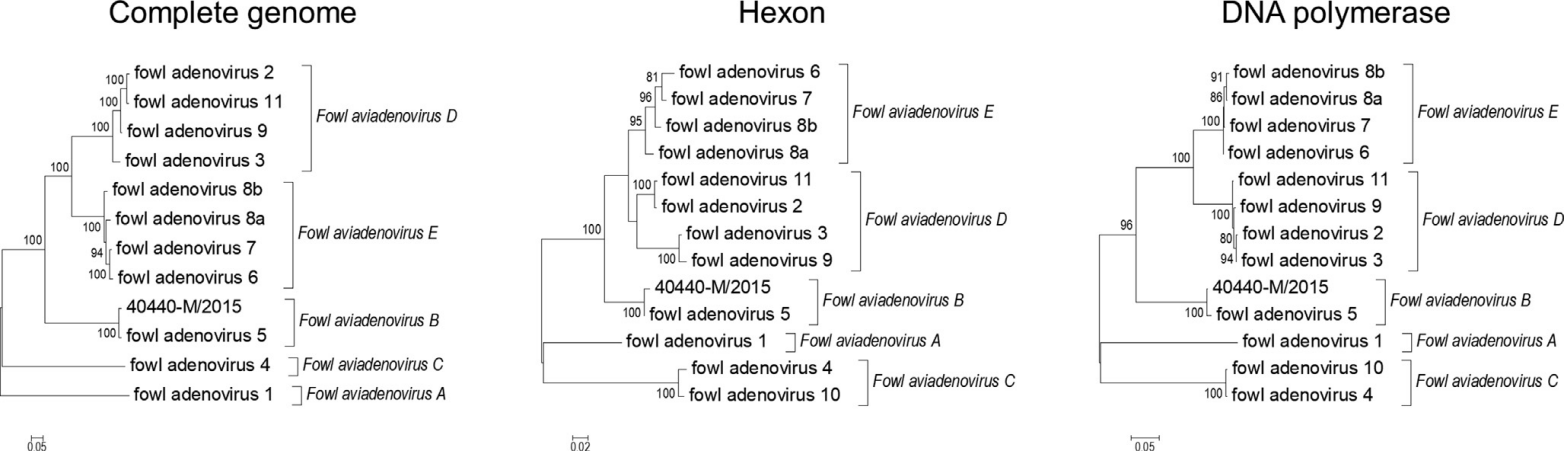
Phylogenetic analysis of strain 40440-M/2015, a putative new fowl adenovirus type within species *Fowl aviadenovirus B*. The analyses were conducted based on three stretches: complete genome sequences and derived amino acid sequences of the entire hexon and the DNA polymerase.

NGS of strain 40440-M/2015 produced 1,567,050 reads and the complete genome sequence was found to be 45,743 bp long with a G + C-content of 56.4%. The final read coverage minimum was 46 (in the first intragenomic repeat region), the mean was 3349.7, the read coverage's standard deviation was 991.4, and the average of the base quality sums was 105,198.1. A typical FAdV genome layout was observed with 36 protein coding sequences and 82-bp-long inverted terminal repeats (ITRs) (Fig. 2). The genome sequence showed an overall 97.44% of sequence identity with strain 340. The regions exhibiting the lowest identity were the genes of capsid proteins, ORF19 and the repeat regions (Fig. 2). The results of the pairwise sequence comparisons – the SDT analyses – are summarised in Table 2 and the respective phylogenetic trees are presented in Fig. 3.

**Table 2:** Pairwise sequence identity analysis of strain 40440-M/2015.

Analysed stretch	FAdV serotypes with the highest sequence identity between them			Other FAdV type pairs with higher sequence identity between them than measured between strains 40440-M/2015 and 340	Sequence identity between strains 40440-M/2015 and 340 (FAdV-5)
	Serotype ordinal No	FAdV species	Identity		
Complete genome (nt)	2 & 11	D	97.98%	-	97.44%
DNA polymerase (aa)	7 & 8a	E	99.69%	11 pairs^a^	98.61%
Penton base (aa)	4 & 10	C	100.00%	6 & 8b; 2 & 11; 7 & 8a	99.63%
Hexon - complete (aa)	2 & 11	D	97.79%	3 & 9; 4 & 10	97.27%
Hexon - loop 1 (aa)	4 & 10	C	92.55%	2 & 11; 6 & 7; 3 & 9	89.94%
Fiber knob (aa)	4 ​F1 & 10 ​F1^b^	C	96.67%	-	92.11%
Concatenate (aa)^c^	2 & 11	D	96.75%	4 & 10	96.19%
ORF19 (nt)	6 & 7	E	99.77%	9 & 11; 2 & 11; 2 & 9; 6 ​& ​8a; 7 & 8a	79.61%
ORF19 (aa)	6 & 7	E	99.59%	9 & 11; 2 & 11; 2 & 9; 6 ​& ​8a; 7 & 8a	83.82%

Abbreviations: aa: amino acid; FAdV: fowl adenovirus; nt: nucleotide; ORF: open reading frame.

a The 11 pairs: 4 & 10; 2 & 3; 6 & 7; 2 & 9; 9 & 11; 8a & 8b; 3 & 11; 6 & 8a; 7 & 8b; 3 & 9; 2 & 11.

b Fiber 1 of fowl adenovirus 4 and 10.

c Concatenate of the major antigenic determinants: penton base, hexon loop 1, hexon loop 2 and fiber knob amino acid sequences. Both fiber 1 and 2 were included in the concatenate for FAdV-1, -4 and -10.

The new sequences were submitted to the NCBI database. The genome sequence of strain 40440-M/2015 was assigned to accession number MG953201. The partial hexon gene sequences were deposited under accession numbers MG953202–MG953229.

## 4 Discussion

The PCR- and sequencing-based molecular typing of the new AdV isolates confirmed the presence of all five FAdV species in Eastern Hungary. This finding is in accordance with the results of a previous screening (Kaján et al., 2013). Similarly, the majority of the new AdV strains were also found to belong to species FAdV-D and -E. Interestingly, from species FAdV-D only FAdV-2 was isolated now. In our previous study, FAdV-11 had been dominant, but FAdV-2 and -3 had occurred as well. From species FAdV-E, 8a was the most prevalent type, followed by 8b, but no FAdV-7 was isolated this time. Similarly, no isolates representing types FAdV-3 or -11 were obtained, though their circulation has been described in Hungary previously.

In Italy, a screening revealed that all FAdV species were present, except FAdV-B, and most often (in 41% of the samples) strains belonging to species FAdV-E were found (Pizzuto et al., 2010). In Poland, isolates representing all the five FAdV species have been reported, including FAdV-11 from species FAdV-D (Niczyporuk, 2016) too, which we could not detect at all in our samples. The majority of the isolates obtained in Belgium have been found to belong to various types of species FAdV-D and -E (De Herdt et al., 2013). Outside Europe, the picture changes slightly only. Various types belonging to species FAdV-D, -E or -A have been reported from Canada (Ojkić et al., 2008), whereas FAdV-8b and -11 have been found to be the most common in Australia (Steer et al., 2011). FAdV-2 (from species FAdV-D) has been described as the predominant type in Japan (Nakamura et al., 2011). In the continental Asia however, FAdV-4 and HHS occur more commonly. FAdV-3, -4, -8b, -9 and -11 have been detected in Korea (Choi et al., 2012; Lim et al., 2011). HHS cases, linked to FAdV-4, as well as IBH cases associated with FAdV-8a, -8b and -11 infection have been reported from China (Wang et al., 2018).

The pathological findings indicated that most of the cases were not the result of a simple FAdV infection (Table 1). Besides pathogenic strains of FAdV-1, a FAdV-2 strain (3792-M/2013) from species FAdV-D, was also isolated from GE. An additional FAdV-1 isolate was obtained from a case of gizzard atrophy. Indeed, GE is usually linked with FAdV-1 (De Herdt et al., 2013; Domanska-Blicharz et al., 2011; Grafl et al., 2012; Kecskeméti et al., 2012; Lim et al., 2012; Schade et al., 2013; Thanasut et al., 2017), though Mase and Nakamura isolated four FAdV-E strains from the same clinical manifestation (2014). On the other hand, since concurrent infections with multiple FAdV types are common (Kaján et al., 2013), it cannot be excluded that the FAdV-2 strain 3792-M/2013 originated from a sample that also contained FAdV-1.

In connection with IBH cases, we isolated FAdV-2 and -8b strains exclusively. This confirms the recent results (Schachner et al., 2016), according to which only specific (FAdV-2, -8a, -8b or -11), but not all types from species FAdV-D and E, could be associated with IBH cases. We isolated the new FAdV-2 strain (7472-M/2012) from a case where IBH was diagnosed in all the ten dead birds submitted for examination. Nonetheless, not every Hungarian FAdV-D/E isolate was obtained from typical IBH cases.

Certain pathological findings were attributed to the presence of various additional pathogens, as concurrent infections were found in almost every case, we studied. Among the most common lesions were fibrinous airsacculitis and pericarditis (7 cases), always accompanied by *Escherichia coli* infection. Catarrhal or necrotising enteritis were also detected mainly in connection with *Escherichia coli* (11 cases), or sometimes with reoviruses (6 cases). Upper respiratory tract inflammation was found in birds positive for infectious bronchitis virus (3 cases). Among other infections, we diagnosed chicken anaemia virus (2 cases) and a resolved case of infectious bursal disease. One bird suffered from coccidiosis as well. These findings indicate that concomitant infections may contribute to the mortality in various extent. It has been described that infectious bursal disease and chicken anaemia virus are predisposing factors in FAdV infected flocks for clinical manifestation of IBH. It has also been demonstrated that avian reoviruses can increase the pathogenicity of at least some of the infectious agents in chickens (De Herdt et al., 2013).

Based on the phylogenetic analysis of the partial hexon aa sequences, six strains were classified in species FAdV-B. There is only one accepted serotype within this species, namely FAdV-5, represented by reference strain 340 (Marek et al., 2013). It is notable that only one isolate (9892-M/2013), had 100% aa sequence identity with the reference strain, while the remaining five strains shared only 91.4% (data not shown) but clustered together with it (Figs. 1 and 3). A similar finding has been reported in the previous Hungarian study, where three of the novel FAdV-B isolates could not be classified into type FAdV-5, because, on the examined gene portion, they shared only 90.9–91.9% nt identity with strain 340 (Kaján et al., 2013). Even before this, an analogous situation has been published by Marek et al. (2010) in Austria. Interestingly, four from the five Austrian FAdV-B isolates were identical on the nt level with the divergent Hungarian strains. To analyse such a strain thoroughly, we set out to determine the complete genome sequence of one of them (40440-M/2015).

In every pairwise-alignment-based SDT analysis (Table 2), a threshold was determined first. This threshold was the sequence identity percentage measured between the two closest related FAdV serotypes (more specifically their reference strains) in that specific analysis. E.g., FAdV-2 and -11, two distinct serotypes in species FAdV-E, show 97.79% sequence identity based on the derived aa sequence of the major antigenic determinant, the hexon protein. This percentage value was used as a threshold for the analysis. In each analysis, strain 40440-M/2015 diverged from FAdV-5 (strain 340) by a higher degree than the threshold value. In other words, it shared lower sequence identity with FAdV-5. In seven out of the nine analyses conducted, not a single pair of FAdV serotypes showed higher sequence identity to each other than that between strains 40440-M/2015 and 340. E.g., in the analysis of the sequences of the viral DNA polymerase, twelve pairs of FAdV serotypes showed such, higher sequence identity to each other. Based on these diverging sequence identity results, we think that demarcation of these novel strains from type FAdV-5, within the species FAdV-B, would be justifiable. These strains obviously represent a separate FAdV genotype as has been proposed repeatedly previously by Marek et al. (2010) and by us as well (Kaján et al., 2013). Because of the high and ever growing number of different AdV types, serum neutralisation is no longer a routine practice in diagnostic laboratories. Instead, molecular genetic methods are tested as appropriate alternatives for typing. The novel isolates characterised by sequence data are usually called just types or genotypes, although the more sophisticated SDT methods are likely capable of demarcating virus strains that represent distinct *sero*types indeed.

The genome organisation of strain 40440-M/2015 was found to be almost identical to that of strain 340, the prototype strain of FAdV-5 (Marek et al., 2013). Merely three, minor open reading frames (ORF29, ORF29A and ORF29B) were left out from the annotation. They seemed rather to be just short repeat regions (nucleotides 39,128–39,249 in strain 40440-M/2015) and not putative genes. This short genomic stretch contained two identical repeats (39,179-39,204 and 39,224-39,249) and several variations. Between the two genomes, the highest level of sequence divergence was observed in the sequence of ORF19 (Fig. 2). The extent of both the aa and nt sequence divergences between strains 40440-M/2015 and 340 on this ORF (Table 2) are already close to interspecies divergence levels (e.g., comparable to that between FAdV-3 [FAdV-D] and FAdV-8b [FAdV-E]: aa sequence identity: 80.83%; nt sequence identity: 78.50%). The predicted product, coded by ORF19, is a membrane protein that is homologous to the lipase of the Marek's disease herpesvirus, in which it is a virulence factor (Davison et al., 2003; Kamil et al., 2005; Tulman et al., 2000). This putative gene is preserved in almost every FAdV type but is missing or truncated in several, highly pathogenic FAdV-4 strains (Griffin and Nagy, 2011; Pan et al., 2017a, 2017b). Nonetheless, the exact role of ORF19 in the life cycle or the pathogenicity of FAdVs needs further investigation. Because of their repetitive nature and the presence of the terminal protein covalently attached to the genome ends (Rekosh et al., 1977), the sequencing of adenoviral ITRs might be challenging, sometimes requiring additional amplification (Kaján et al., 2012). For this genome, no additional work was needed as the read coverage minimum was 1,410 in these regions and the average of base quality sums were 29,610.2 and 81,090.0 in the region of the right- and left-hand ITRs, respectively. Furthermore, the newly-determined sequences were in good agreement with that of strain 340, which possesses ITRs encompassing of 86 nucleotides.

The literature regarding the pathogenicity of FAdV-B strains is scarce. FAdV-5 isolates have been obtained from bantam chickens that had died suddenly, as well as from healthy mallards and from swollen tarsal joints of lame chickens (Marek et al., 2010; McFerran et al., 1976). The novel strains, proposed to be classified as a new genotype were associated with numerous pathological lesions: in one case with IBH (Marek et al., 2010), later with airsacculitis, pericarditis and enteritis (Kaján and Kecskeméti, 2011; Kaján et al., 2013), and now with different cases of cardiac decompensation, enteritis and nephrosis, among others. The exact pathological role of the FAdV-B strains is yet unclear. Further investigations are needed to broaden our knowledge about the pathogenicity of the virus and the eventual prevention of the infection.

## 5 Conclusions

The current study showed that all FAdV species can be found in Hungary and are involved in diseases of chickens alone or, more frequently, in association with other infectious agents. An emerging new FAdV genotype (belonging to species FAdV-B) is already present in Hungary and Austria, though clarification of its exact pathological role requires further investigations. Our research inserts itself in a global work of FAdV screening and we hope that our results will be useful in the epizootiological investigation of FAdV infections, or for the purpose of vaccination against FAdVs, *in primis* in Central Europe.

## Declarations

### Author contribution statement

Győző L. Kaján: Conceived and designed the experiments; Performed the experiments; Analyzed and interpreted the data; Contributed reagents, materials, analysis tools or data; Wrote the paper.

Ilaria Affranio: Performed the experiments; Analyzed and interpreted the data; Wrote the paper.

Andrea Tóthné Bistyák, Sándor Kecskeméti: Performed the experiments; Contributed reagents, materials, analysis tools or data.

Mária Benkő: Contributed reagents, materials, analysis tools or data; Wrote the paper.

### Funding statement

This work was supported by the National Research, Development and Innovation Office, Hungary (grant NN 128309) and the OMA Foundation, Hungary (grant 101öu6).

### Competing interest statement

The authors declare no conflict of interest.

### Additional information

No additional information is available for this paper.
